# Benefits of Adopting Hypofractionated Radiotherapy as a Standard of Care in Low-and Middle-Income Countries

**DOI:** 10.1200/GO.22.00215

**Published:** 2022-12-16

**Authors:** Ryan D. Kraus, Christopher R. Weil, May Abdel-Wahab

**Affiliations:** ^1^ Department of Radiation Oncology, Huntsman Cancer Institute, University of Utah, Salt Lake City, UT; ^2^ Division of Human Health, International Atomic Energy Agency, Vienna, Austria

## INTRODUCTION

Globally, cancer is the leading cause of mortality and accounted for approximately one in six deaths in 2020.^1^ The burden of cancer mortality falls disproportionately onto low-and middle-income countries (LMICs) which have higher cancer mortality-to-incidence ratios.^2,3^ Many factors contribute to this disparity, including limited access to treatments, more frequent advanced stages of disease at presentation, insufficient numbers of trained physicians, and patients being unable to complete their entire planned course of therapy.^2,4,5^ This has led to many international organizations advocating for increasing investments in health care infrastructure in LMICs, with an estimated investment of $184 billion US dollars (USD) required.^2,6,7^ Less emphasis has been placed on how LMICs can more efficiently use the resources already available to them to increase availability and access to high-quality cancer care, complete planned treatments, and improve oncologic outcomes. One avenue to explore is the adoption of hypofractionated radiotherapy regimens for some of the most common cancers.

**CONTEXT**

**Key Objective**
How would the adoption of hypofractionated radiotherapy regimens in low-and middle-income countries (LMICs) affect access to radiotherapy, treatment compliance, and the costs borne by patients and the health care system?
**Knowledge Generated**
The benefits of adopting hypofractionated radiotherapy regimens in LMICs include higher rates of treatment compliance, decreased financial toxicity for patients, decreased costs for health care systems, and improved access to radiotherapy.
**Relevance**
In LMICs, improved treatment compliance and access to radiotherapy achieved through the adoption of hypofractionated radiotherapy regimens has the potential to narrow the disparate oncologic outcomes observed between high-income countries and LMICs. A multifaceted approach, including investments in radiotherapy infrastructure, clinician and physicist-directed training programs, implementation research, and advocacy by stakeholders and global partners, will be needed to overcome the infrastructure and knowledge gaps which currently prohibit widespread adoption of hypofractionated treatment regimens in LMICs.

Hypofractionated radiotherapy is a treatment approach that shortens the overall duration of a radiotherapy treatment course by delivering fewer treatments but with a higher dose of radiation per daily treatment. These hypofractionated treatments have become the standard of care for patients with breast and prostate cancer in the United States and Europe, as multiple studies have shown that they provide noninferior oncologic outcomes and have a similar toxicity profile but can be delivered in a shorter period of time at a lower cost to the health system.^8-14^ This has enabled radiotherapy courses for patients with breast cancer to be shortened from 5-6 weeks to 3-4 weeks or even 1 week.^8,11,12^ Similarly, prostate cancer treatments have decreased from 8-9 weeks in length to 4-6 weeks or 1 week in certain circumstances.^9,10,13^ Additionally, hypofractionated approaches for lung, rectal, and liver cancer have become established treatment options.^15-19^ Data are also accruing supporting the use of hypofractionated radiotherapy for head and neck and gynecologic cancers.^20-25^ More efficient utilization of resources through the adoption of hypofractionated radiotherapy approaches for some of the most common malignancies, particularly breast and prostate cancer, has the potential to address many of the factors contributing to the disparity in cancer outcomes seen between LMICs and high-income countries (HICs). In this literature review, we aim to examine the potential benefits of adopting hypofractionated treatment approaches in LMICs and the current state of hypofractionated radiotherapy in these settings.

## METHODS

An exhaustive review of available literature was performed using the PubMed database. Publications pertaining to hypofractionated radiotherapy and cost-effectiveness, treatment compliance, or treatment access were included for review. Full-text papers published in English between 2000-2022 were initially identified through a PubMed search including the Mesh terms "Radiation” [Mesh] and (“Hypofractionation” [Mesh] OR “Hypofractionated” [Mesh]) AND (“Accessibility” [Mesh] OR “Access” [Mesh] OR “Cost” [Mesh] OR “Compliance” [Mesh] OR “Completion” [Mesh] OR “Benefit” [Mesh]). The initial papers identified were then back-referenced to identify additional relevant studies. Publications were included in this review if they addressed hypofractionated treatment regimens in LMICs. A review of US National Library of Medicine using the search terms “hypofractionated,” “SBRT,” “stereotactic,” “ultrahypofractionated,” “fractionated,” “moderately fractionated” was performed to identify clinical trials using hypofractionated radiotherapy regimens.^26^

### Improved Access to Radiotherapy

Access to radiotherapy in many regions is often limited because of an insufficient number of radiation oncology clinics, trained personnel, and treatment machines.^2,4,27,28^ The consequences of limited availability of radiotherapy are best illustrated by a report from Brazil in 2016 which found that limited access to radiotherapy was estimated to result in more than 5,000 deaths among patients with prostate, breast, colorectal, lung, and cervical cancer.^29^ In a 2020 analysis of 46 countries, it was estimated that radiotherapy is only accessible to approximately 62% of the patients who could benefit from it and that an additional 5,987 treatment machines would be needed in LMICs to fully meet the radiotherapy demand.^27^ Accessibility varies by region, with one report indicating that access to radiation was lowest in Africa (34%), followed by Asia-Pacific (61%) and Latin America (88%).^4^ Clearly, additional investment in infrastructure is needed, but the required investment can be reduced with optimal utilization of radiotherapy through hypofractionation, as shown in Table 1. Adopting hypofractionated treatment approaches alone was estimated to improve access to radiotherapy in Asia from 62% to 78% and decrease the number of treatment machines needed in LMICs from 5,987 to 4,284, significantly reducing the investment required to improve access to radiotherapy.^27^ Another published report estimated that implementing universal hypofractionated treatments for breast and prostate cancer would increase access to radiotherapy in Africa by up to 25% for breast cancer and 36% for prostate cancer.^30^ Using Nigeria as an example, which had seven linear accelerators (LINACs) in 2020, it is estimated that the increase in patient throughput with the adoption of ultrahypofractionated prostate radiotherapy would allow all eligible patients with prostate cancer in the country to receive treatment without increasing the number of LINACs.^31^ Similar results were seen when assessing the impact of adopting moderately hypofractionated radiotherapy for prostate cancer in Brazil.^31^

**TABLE 1:**
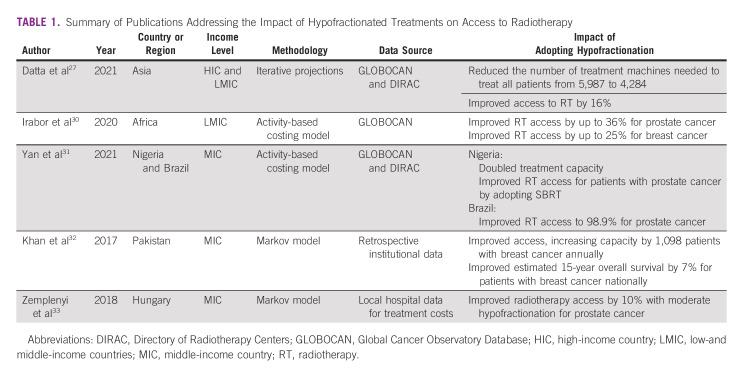
Summary of Publications Addressing the Impact of Hypofractionated Treatments on Access to Radiotherapy

Condensing the overall treatment time with hypofractionation allows each LINAC to treat more patients per year, with treatment slots becoming available more frequently. Patients who otherwise may have to wait multiple weeks for a treatment slot can then be accommodated in a more timely fashion, preventing the delays in initiation of radiotherapy that have been associated with worse outcomes.^34,35^ A Markov model looking at the clinical implications of widespread adoption of hypofractionated radiotherapy for breast cancer in Pakistan, which in 2017 only had 15 LINACs for a population of 180 million people, estimated that an additional 1,098 patients with breast cancer could be treated per year if hypofractionation was the standard of care.^32^ This translated to an estimated 7% 15-year overall survival benefit among patients with breast cancer in Pakistan illustrating how improved access to radiotherapy through adoption of hypofractionated treatments can lead to improved oncologic outcomes.

### Hypofractionation and Treatment Compliance

Improving access to radiotherapy is the first key step to eliminating the disparities in oncologic outcomes between LMICs and HICs. However, if the radiotherapy courses being offered cannot be feasibly completed by patients, the clinical benefits of improved access will be limited. Two of the most cited risk factors for patients being either unable to complete treatment or having treatment interruptions are prolonged treatment courses and lower socioeconomic status.^36-38^ Both of these risk factors may be mitigated by hypofractionated radiotherapy treatment approaches.

Reports on the incidence of treatment interruptions vary on the basis of the primary site and the treatment setting, but reports range from 20% to 71% while the incidence of treatment discontinuation range from 13% to 61%.^36,38-45^ Compliance with a planned radiotherapy course is crucial as multiple studies have shown that treatment delays and interruptions are associated with worse oncologic outcomes.^34,35^ The impacts of treatment interruption or discontinuation also have knock on effects on available resources. For instance, treatments missed may need to be made up at the end of the treatment course, further prolonging treatment. Patients with very protracted or incomplete treatments have a higher risk of recurrence, which could result in the need for additional radiotherapy treatment courses in the future, further straining resources.^35^ Treatment delays and treatment discontinuation can occur because of clinic-based factors (machine downtime, understaffed centers being unable to accommodate all patients, or insufficient medical supplies or resources) or for patient-based reasons (financial, logistical, or personal). In regard to patient-specific factors, a shorter treatment course can make compliance with a planned radiotherapy course more feasible by reducing housing and transportation costs, and decreasing time away from work and family.^45^ Unfortunately, there is currently no data available quantifying the impact hypofractionated radiotherapy may have on treatment compliance in LMICs.

However, there are some limited data on this topic as it relates to breast cancer in the setting of HICs, as shown in Table 2. A study from Saudi Arabia analyzed the factors affecting treatment interruptions in a population of 286 patients receiving postoperative radiotherapy for breast cancer, of whom half received a hypofractionated 3-4.5 week course and half received a standard 5-7 week course, with length depending on inclusion of a lumpectomy cavity boost.^40^ Overall, 20% of patients had a treatment interruption of at least one day, but patients treated with conventional fractionation were twice as likely to have a treatment interruption (27% *v* 14%, *P* = .007). Additionally, patients treated with conventional fractionation had significantly longer treatment interruptions (3 *v* 2 days, *P* = .02) compared with patients treated with a hypofractionated course.

**TABLE 2:**
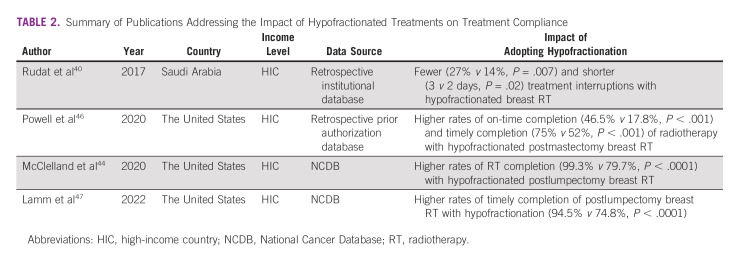
Summary of Publications Addressing the Impact of Hypofractionated Treatments on Treatment Compliance

Three studies from the United States, including one institutional study and two National Cancer Database (NCDB) studies, reported similar findings.^44,46,47^ The institutional study reported on 743 patients with breast cancer, 56 of which were treated with hypofractionated therapy and reported on rates of on-time completion (defined as treatment completion assuming treatment is given 5 days per week with an additionally 7-day buffer) and timely completion (similar to on-time but with a 30-day buffer) of radiotherapy. Hypofractionation was associated with higher rates of on-time completion (46.5% *v* 17.8%, *P* < .001) and timely completion (75% *v* 52%, *P* < .001) of radiotherapy.^46^ The two NCDB studies were consistent with these results and reported higher overall completion rates (99.3% *v* 79.7%, *P* < .0001) and timely completion rates (94.5% *v* 74.8%, *P* < .0001) which was defined as treatment completion within 5 weeks of initiation of hypofractionated radiotherapy or 7 weeks for conventionally fractionated treatments.^44,47^ Importantly, racial and socioeconomic disparities in treatment completion rates and tumor control were narrowed when a hypofractionated radiotherapy approach was used because of higher rates of treatment compliance and completion.^47^ This gives some hope that the implementation of hypofractionated radiotherapy may allow more patients to complete their recommended treatment course and help to narrow the differences in cancer mortality-to-incidence ratios between LMICs and HICs.

### Reduced Costs

Multiple studies have shown that hypofractionated radiotherapy for patients with breast and prostate cancer is the most cost-effective radiotherapy regimen.^48-52^ Although dependent on a country's health care system, reimbursement often scales with the number of fractions, making fractionation the largest contributing factor to the cost of radiotherapy treatments.^50^ However, most cost-effectiveness data have been reported from HICs, with only five studies focused on LMICs, as shown in Table 3. The first of these studies reported that reducing breast cancer treatments from 25 to 15 fractions across Africa would save an estimated $1.1 billion USD between 2019 and 2025 while reducing prostate cancer treatments from 39 to 20 fractions would save an additional $606 million USD over the same time period.^30^ This represents a significant amount of capital which could then be invested in health care infrastructure.

**TABLE 3:**
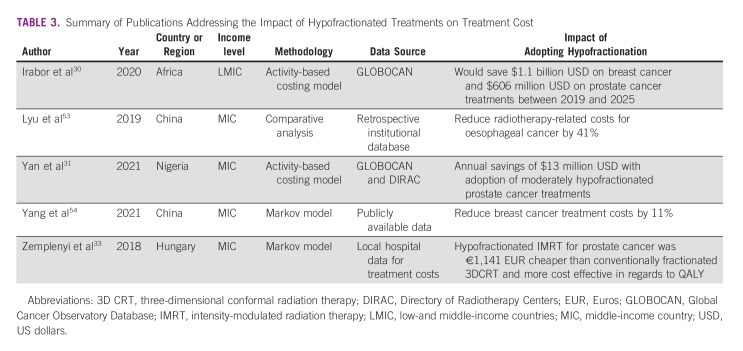
Summary of Publications Addressing the Impact of Hypofractionated Treatments on Treatment Cost

Four country-level studies have evaluated cost-effectiveness. The first reported that 25-fraction moderately hypofractionated intensity-modulated radiation therapy (IMRT) for prostate cancer in Hungary was more cost-effective (absolute savings of €1,141 Euros) than a 35-39-fraction three-dimensional radiation therapy (3DCRT) course, despite the extra planning and technology costs and requirements associated with IMRT.^33^ Additionally, moderately hypofractionated radiotherapy resulted in a 10% increase in the number of patients who could be treated because of to increased machine capacity.^33^ Adoption of moderately hypofractionated radiotherapy for prostate cancer was predicted to save the Nigerian health care system approximately $13 million USD annually after accounting for required LINAC upgrades to deliver IMRT and image-guided radiation therapy (IGRT), consumable resources, construction, machine maintenance, and educational and personnel costs.^31^ A study on the cost effectiveness of hypofractionated compared with conventionally fractionated postmastectomy breast radiation in China found that it was associated with an 11% cost reduction and was determined to be cost-effective.^54^ Another study from China reported that neoadjuvant 10-fraction hypofractionated radiation for esophageal cancer resulted in a 41% reduction in radiotherapy costs over a 20-fraction moderately hypofractionated regimen.^53^

Reducing the treatment costs patients incur is equally important to reducing the costs for the overall health care system. The cost associated with traveling, finding housing, and missing work can carry staggering consequences for patients. For instance, the majority of Nigerian patients with breast or cervical cancer reported a moderate or major loss of revenue because of not being able to work (68%) with one third of patients also reporting that their family members had moderate or major losses in revenue (32%).^55^ Given these financial challenges, it is not surprising that 23% of Nigerian patients from this cohort reported taking out loans to cover the cost of their medical care and daily needs. Similar challenges were faced by Argentinian patients with cervical cancer who experienced reduced hours worked (45%), more work interruptions (25%), a loss of family income (39%), reduced amounts of food consumed by their family (37%), delays in paying for essential services such as electricity (43%), the sale of property or use of savings to cover basic need (38%), and disruptions in children's schooling (28%).^37^ This highlights how the burden of prolonged treatments can negatively affect entire families. Additionally, patients who lost household income as a result of their cancer treatments were less likely to be compliant with their scheduled radiotherapy, further hinting at the interplay between socioeconomic factors and oncologic outcomes.^37^

There are minimal data quantifying how using shortened hypofractionated treatment courses would affect a patient's out-of-pocket expenses in LMICs. A study found that when accounting for traveling expenses alone, Canadian patients had an additional $1,930 Canadian dollars of out-of-pocket expenses when treated with a 39-fraction radiotherapy regimen for prostate cancer compared with a 5-fraction regimen.^56^ This is further supported by an American study which reported that the cost to the patient in nonmedical expenses was approximately 50% less when using a 16-fraction treatment approach compared with a 25-fraction treatment approach for breast cancer, largely because of decreased traveling expenses and lost wages because of daily travel requirements.^57^ However, even this study does not fully account for either the cost of prolonged periods of missed work when patients must relocate to receive radiotherapy or the lost productivity after completion of treatment, which may account for approximately one third of the true economic cost of a patient's cancer treatment.^58^ Despite the lack of data from patients in LMICs, these findings are likely applicable to patients in these settings as a shortened treatment course decreases the need for housing, time away from work, and minimizes expenses related to traveling for daily treatments. These benefits may not be accounted for when assessing the value of hypofractionated radiotherapy from a health care system's perspective but are likely highly valued by patients.

### State of Hypofractionation in LMICs

Despite the benefits associated with hypofractionated radiotherapy, the adoption rate of hypofractionated treatments has varied widely between countries. A recently published European Society for Radiotherapy and Oncology's Global Impact of Radiotherapy in Oncology (ESTRO-GIRO) survey, completed by 2,316 radiation oncologists around the world, evaluated adoption rates of hypofractionated radiation for bone metastases and breast, prostate, and cervical cancer.^59^ They found that while hypofractionation was widely adopted for palliative radiotherapy, accounting for approximately 75% of palliative treatments, the utilization for definitive indications varied significantly by region. For example, low-risk prostate cancer was treated with hypofractionated radiotherapy at higher rates in North America (94%) compared with Europe (67%), Latin America (44%), Asia-Pacific (42%), Middle East (31%), and Africa (19%). Although the absolute difference in utilization between these regions decreased for intermediate-and high-risk prostate cancer, the disparate utilization rates still persisted. Similar trends were seen for node-negative postlumpectomy breast cancer, with hypofractionated regimens being more common in North America (97%) compared with Europe (89%), Latin America (77%), Middle East (76%), Asia-Pacific (72%), and Africa (40%).

**FIG 1:**
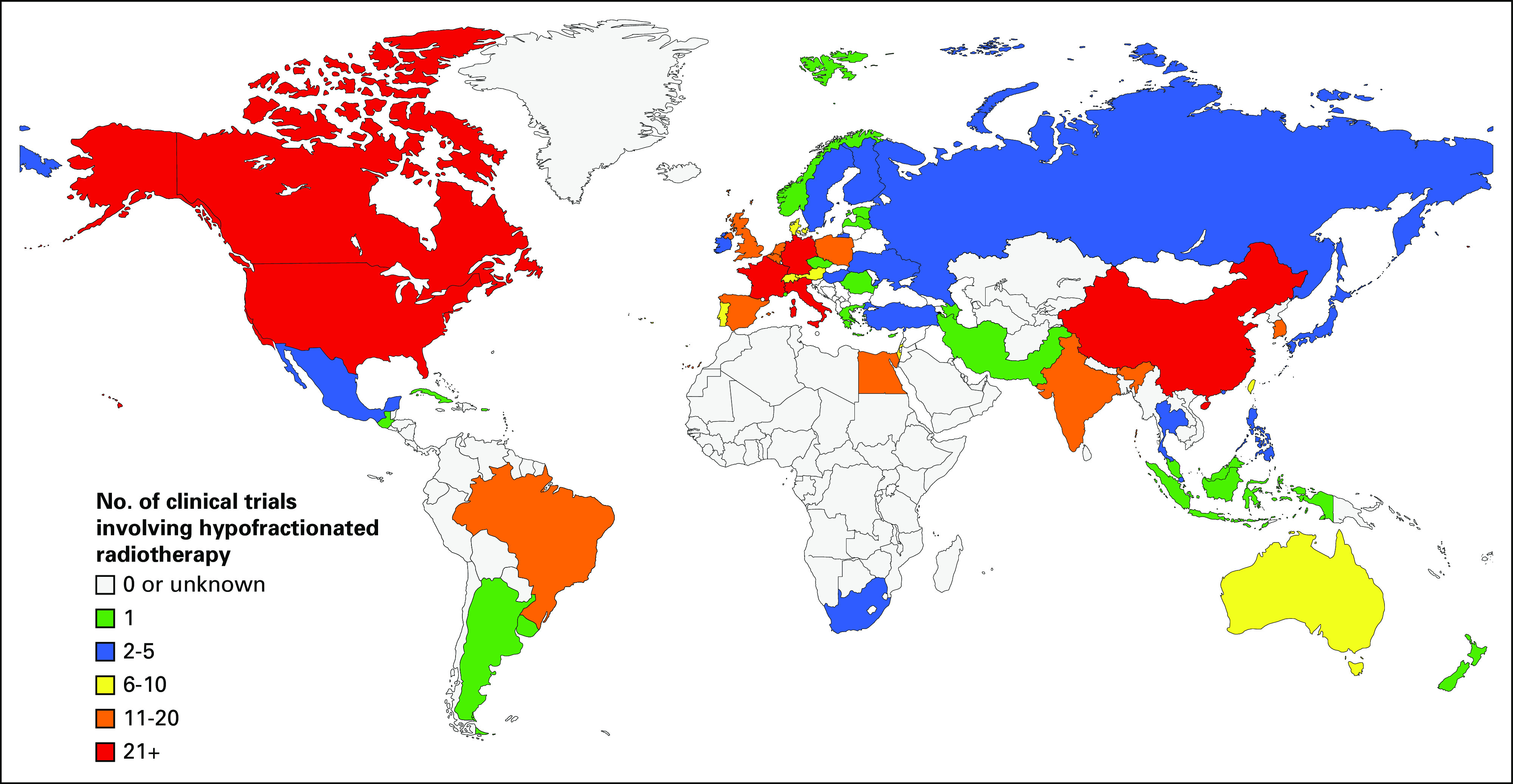
World map of countries color coded on the basis of the number of ongoing clinical trials involving hypofractionated radiotherapy regimens.^60^

To assess the current state of hypofractionated radiotherapy utilization across the world, we analyzed which countries had ongoing clinical trials involving hypofractionated radiotherapy using the US National Library of Medicine database, as shown in Figure 1. The majority of the trials involving hypofractionation are being performed in HIC (82%), followed by middle-income countries (12%), with no registered ongoing clinical trials in low-income countries.^26^ Breast, prostate, and bladder represented the majority of the disease sites. Of the LMICs doing these clinical trials, the largest portion of these are being done in China (46%), Brazil (13%), and India (9%).

These hypofractionated approaches have increasingly been recommended by professional societies since the COVID-19 pandemic as a way to limit patient's risk of infection by decreasing their exposure to the medical system.^21,61,62^ Early reports indicate that radiation oncologists from LMICs have increased their utilization of hypofractionated regimens since the beginning of the COVID-19 pandemic with one report from India finding that 9% of radiation oncologists had implemented hypofractionated treatments for the first time.^63-66^ Similarly, multiple institutions have reported that patients on average are being treated with fewer fractions per treatment course since the beginning of the COVID-19 pandemic, suggesting increased adoption of hypofractionated treatment regimens.^67,68^

Despite these recent advances in the adoption of hypofractionated radiotherapy regimens, the results of the ESTRO-GIRO survey indicate that the regions that would most benefit from the widespread adoption of hypofractionated treatments also have the lowest utilization rates. The reason why hypofractionation is not more widely used varied by region, but surprisingly, technology was only cited as a significant barrier by 24% of respondents from Latin America, 23% of respondents from the Middle East, 19% of respondents from Africa, 16% of respondents from Asia-Pacific, 11% of respondents from Europe, and 3% of respondents from North America.^59^ Most respondents were more likely to cite a lack of long-term data (18%-61%), fear of inferior local control (14%-32%), or concern for increased toxicity (23%-56%) as being significant barriers to adoption. This may indicate that a knowledge gap exists surrounding hypofractionation, limiting implementation.

Although less than a quarter of ESTRO-GIRO survey respondents reporting that technology represented a barrier to implementing hypofractionated treatments, there is a well-documented technological gap between the radiotherapy resources available in LMICs and HICs. This technology and infrastructure gap likely contributes to the disparity in hypofractionation utilization as the infrastructure, technological capabilities, and expertise required to deliver hypofractionated treatments is not insubstantial, as shown in Table 4. It has been postulated that the minimum requirements for implementing hypofractionated radiotherapy include a LINAC capable of delivering 3DCRT (minimum 10 MV beam) with 5-mm multileaf collimators, computed tomography (CT) treatment simulation (maximum 3-mm slice thickness), forward and inverse treatment planning systems, appropriate immobilization devices, a regimented quality assurance protocol, and well-trained radiation oncologists, physicists, radiation therapists, and dosimetrists.^31,69,70^ Although these represent minimal standards, treatment with IMRT (minimum 6 MV beam) with image guidance and motion tracking systems is preferred but requires additional equipment and expertise. In Brazil's radiotherapy expansion project, the estimated cost to upgrade LINACs to deliver IMRT, including licensing costs, was $350,000 USD, with an additional estimated $350,000 USD to upgrade LINACs to provide IGRT capabilities with cone beam computed tomography.^31^ However, institutions will also face recurring maintenance and software licensing costs, further driving up the long-term investment required to acquire and maintain the infrastructure required to delivery hypofractionated radiotherapy. Costs for maintenance contracts should be factored into the initial investment costs and budget impact analyses.

**TABLE 4:**
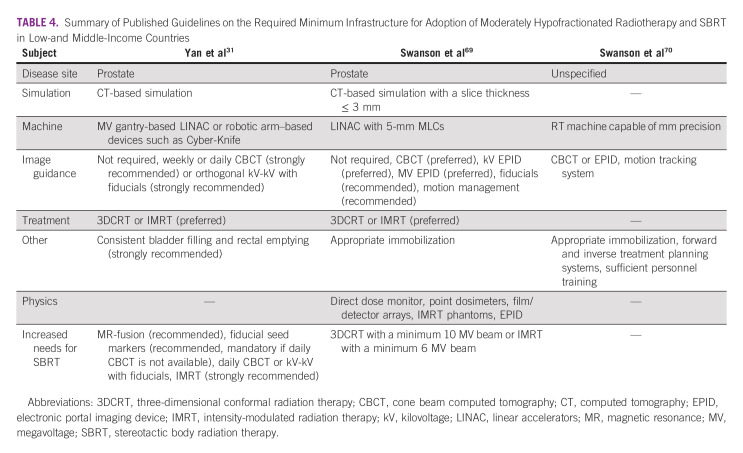
Summary of Published Guidelines on the Required Minimum Infrastructure for Adoption of Moderately Hypofractionated Radiotherapy and SBRT in Low-and Middle-Income Countries

Meeting these infrastructure standards may currently be out of reach for many cancer centers in LMICs, as a 2018 survey of middle-income countries revealed that 49% of patients are still treated with two-dimensional techniques and only 3% of patients are being treated with IMRT.^28^ This concern is further supported by a survey of 18 radiation oncology clinics in Africa which found that none of the clinics had advanced motion tracking systems (four-dimensional CT, infrared light-emitting diodes, and speckle-texture projection) and only one clinic used fiducials in their practice.^70^ These 18 clinics had significant differences in their capabilities with five of the 18 clinics reporting being capable of delivering IMRT while five clinics did not have the equipment necessary to perform CT treatment simulations. If these 18 clinics are representative of the region, it may be that delivering safe hypofractionated radiotherapy is only possible at a minority of treatment centers in Africa, one of the regions with the most to gain from its implementation.^4,30^

### Building Capacity for Hypofractionated Treatments

A multifaceted approach will be needed to overcome the infrastructure and knowledge gaps which may be prohibiting more widespread adoption of hypofractionated treatment regimens. The need for investments in infrastructure cannot be ignored. Of particular importance for hypofractionated radiotherapy are the capability to perform a CT treatment simulation, create treatment immobilization devices, deliver IGRT, and perform regimented quality assurance practices.^69,70^ One of the challenges to acquiring and maintaining the necessary infrastructure for hypofractionated radiotherapy is the limited financial resources earmarked for radiotherapy. This is likely due to competing interests, both within the health care system and outside of it. Strong commitment from policymakers and the political will to address this issue is essential.

Although there are significant costs associated with upgrading infrastructure to support delivery of hypofractionated radiotherapy, there is economic justification to support the investment. A recent model developed by the Global Task Force on Radiotherapy for Cancer Control found that for every $1 USD invested in radiation oncology services in LMICs, there was a $2.95 USD return on investment. These results were consistent across all regions and income levels and represent a very compelling economic argument justifying focused investments in radiation therapy infrastructure. Stakeholders will need to work with large organizations, such as the International Atomic Energy Agency, Rays of Hope, and Radiating Hope, or academic institutions in the United States, Europe, or Asia, to ensure that policymakers and government officials are aware of these benefits and advocate for increasing investments in radiotherapy infrastructure.

Facilities in LMICs that already have the necessary resources to provide hypofractionated treatments, but have not yet integrated them into their clinical practice, should consider implementation research to identify how to effectively introduce hypofractionated treatments.^71^ Publishing these implementation studies will create a framework for the adoption of hypofractionated treatments that other LMIC cancer centers can rely on once they develop the infrastructure needed to deliver these treatments. Appropriately implementing this hypofractionated radiotherapy into clinical practice is critical, as attempting to do so without adequate infrastructure or training can compromise treatment outcomes and lead to increased treatment toxicity.^72^ The knowledge gap that exists regarding hypofractionated treatments and the required quality assurance protocols could potentially be bridged through web-based learning programs and conferencing platforms which are currently used in many LMICs through the International Atomic Energy Agency and other international organizations.^73-75^ These web-based programs could give clinicians from LMICs the opportunity to present cases and treatment plans to regional or international physicians who are aware of the local resources available and have experience with hypofractionation.^76,77^ Similar networks should be created for physicists, dosimetrists, and radiation therapists to receive additional training on the appropriate quality assurance protocols needed when delivering hypofractionated treatments.

These training programs will need to be continually updated as novel targeted therapies are brought to market which may interact with radiotherapy and increase the potential toxicity of hypofractionated treatments.^78,79^ There are still significant unknowns regarding the potential for radiosensitization when using targeted therapies, with one survey finding that only 11% of radiation oncologists from the Netherlands felt there was sufficient information and resources available to allow for adequate decision making when combining these two treatment modalities.^80^ Given the inherent uncertainty regarding these multimodality treatments, physicians should be cautious when combining hypofractionated radiotherapy with new targeted therapies.

In conclusion, the potential benefits of hypofractionated radiotherapy for patients and health care systems in LMICs include higher rates of treatment compliance, decreased financial toxicity for patients, decreased costs for health care systems, and improved access to radiotherapy. A number of barriers exist, both in regards to infrastructure and clinician training, that will need to be overcome before achieving more widespread adoption of hypofractionation. Infrastructure and training investments should be directed toward increasing the capacity for hypofractionated radiotherapy, as these treatments have the potential to address some of the most significant factors contributing to the disparate oncologic outcomes between LMICs and HICs. Further study in LMICs is warranted to identify the minimum infrastructure requirements for the safe delivery of hypofractionated radiotherapy and identify effective processes to help build capacity for adoption of hypofractionation in that setting.
